# Early detection of chronic lung allograft dysfunction with spectral and intrabreath oscillometry

**DOI:** 10.1016/j.jhlto.2026.100575

**Published:** 2026-05-04

**Authors:** Anne Fu, Anastasiia Vasileva, Nour Hanafi, Natalia Belousova, Joyce K.Y. Wu, Ella Huszti, Zoltán Hantos, Chung-Wai Chow

**Affiliations:** a Department of Medicine, Temerty Faculty of Medicine, University of Toronto, Toronto, Ontario, Canada; b Pulmonary Function Laboratory, University Health Network, Toronto, Ontario, Canada; c Toronto Lung Transplant Program, Ajmera Multi-Organ Transplant Unit, University Health Network, Toronto, Ontario, Canada; d Thoracic Medicine and Lung Transplant Unit, St Vincent’s Hospital, Sydney, Australia; e Biostatistics Research Unit, University Health Network, Toronto, Ontario, Canada; f Department of Anesthesiology and Intensive Therapy, Semmelweis University, Budapest, Hungary

**Keywords:** lung transplant, oscillometry, chronic lung allograft dysfunction, pulmonary function testing

## Abstract

**Background:**

Chronic lung allograft dysfunction (CLAD) is the main obstacle to long-term survival after lung transplantation. CLAD is defined by a sustained >3-month drop in the forced expiratory volume in 1 sec (FEV_1_) to ≤80% of the baseline value when other causes are ruled out. Oscillometry is a pulmonary function modality that is sensitive to lung mechanics. This study investigated whether oscillometry at the initial ≥20% drop in FEV_1_ is associated with CLAD.

**Methods:**

This cross-sectional study analyzed 384 double lung recipients transplanted between December 2017 and January 2023. Patients who recovered within 3 months after the initial FEV_1_ drop ≥20% (_reversible drop_FEV_1_, n = 25) and those who developed CLAD (n = 49) were matched by duration post-transplant to patients who had not experienced FEV_1_ drop (_stable_FEV_1_, n = 310). We used Wilcoxon tests with Bonferroni correction for pairwise group comparisons, and logistic regression to analyze the strength of associations between oscillometry and CLAD.

**Findings:**

At initial ≥20% FEV_1_ drop, _stable_FEV_1_ patients exhibited significantly better spirometry and oscillometry than CLAD and _reversible drop_FEV_1_ patients. While spirometry could not distinguish between _reversible drop_FEV_1_ and CLAD, oscillometry could. Reactance at 5 Hz (X5) was significantly (*p*<0.05) worse in the CLAD than _reversible drop_FEV_1_ patients and patients with lower X5 had an increased risk of CLAD (OR = 1.90 [1.01-3.57]).

**Conclusions:**

X5 differentiated between patients who developed CLAD from those whose FEV_1_ recovered. Lower X5 (i.e., greater lung stiffness) at time of FEV_1_ drop identifies patients who are more likely to develop CLAD.

**Take home message:**

Oscillometry at time of FEV_1_ drop can distinguish patients who will develop CLAD from those whose FEV_1_ will recover: the more negative the reactance at 5 Hz, the less elastic the lungs, and the higher the likelihood of developing CLAD.

Lung transplantation (LTx) is an established therapy to improve quality of life and survival in patients with end-stage pulmonary diseases. Despite innovations in medical and surgical care, chronic lung allograft dysfunction (CLAD) remains the primary obstacle to long-term survival, with CLAD affecting up to 50% of patients within 5 years of transplant.^1^, ^2^, ^3^

Currently, there is no treatment for CLAD besides re-transplantation. Clinical trials of new therapies are hampered by the retrospective nature of the diagnosis. While CLAD is suspected when forced expiratory volume in 1 s (FEV_1_) drops by ≥20% from the baseline (i.e., highest) value achieved post-transplant, the diagnosis can only be confirmed when the drop is sustained for >3 months after reversible causes, such as acute rejection or infection, have been ruled out.^3^ Baseline FEV_1_ is defined as the average of the 2 highest FEV_1_ measurements post-LTx at least 3 weeks apart.^1^, ^3^, ^4^

Oscillometry is a lung function test that measures the total respiratory impedance to superimposed small-amplitude pressure signals during normal quiet breathing, to provide measurements of respiratory resistance and reactance.^5^ Spectral oscillometry uses multiple-frequency oscillations to estimate the mean impedance values at each frequency of an entire breath. Intrabreath oscillometry uses a single frequency to track the changes in respiratory mechanics continuously during a breath, capturing changes during inspiration and expiration.^5^, ^6^ Oscillometry has benefits over spirometry as it is effort-independent and can be completed in 10 min by anyone who can breathe while wearing a nose clip. This can be advantageous early after LTx due to postoperative pain.

Oscillometry is more sensitive to graft dysfunction when compared to spirometry, the current gold standard tool of monitoring graft function after lung transplant. Oscillometry can detect changes associated with biopsy-proven acute rejection that was undetectable by spirometry.^7^ Variability in oscillometry measured during the first post-transplant year was associated with a higher burden of acute rejection during the year and the future risk of CLAD.^8^ Lastly, oscillometry can discriminate between patients with stable FEV_1_ (_stable_FEV_1_) and CLAD, with different patterns found in patients with bronchiolitis obliterans syndrome (BOS) and restrictive allograft syndrome (RAS)-CLAD phenotypes.^9^

We hypothesized that oscillometry can facilitate the early identification of CLAD by providing risk stratification at the time of the initial drop of 20% in the FEV_1_. The current study investigated whether oscillometry can differentiate whether a patient will recover or develop CLAD at the initial ≥20% drop in FEV_1_.

## Materials and methods

The University Health Network (UHN) Research Ethics Board approved the study (REB# 17–5652). Written informed consent was obtained before assessment. All double LTx recipients were eligible for enrollment at the first outpatient visit to the Toronto General Pulmonary Function Laboratory. We excluded patients who had anastomotic issues, single lung transplant, or remained hospitalized at 3 months post-transplant. Spectral 5-37 Hz multi-frequency oscillometry was followed by 10 Hz oscillometry, a research modality^7^, ^8^, ^9^ with the tremoflo C-100 (Thorasys, Montreal, Canada) following European Respiratory Society guidelines.^10^, ^11^ Oscillometry was performed prior to every spirometry at the Toronto General Pulmonary Function Laboratory. Routine spirometry and plethysmography occur weekly from the time of discharge to the first 3 months post-transplant, then at every 3 months up to 2 years followed by annually thereafter. Patients are also instructed to start daily home spirometry within days after discharge. Spirometry and plethysmography from local pulmonary function laboratories outside of the scheduled visits at UHN, demographic data, and clinical parameters known to affect lung function and graft rejection, including primary lung disease, donor-recipient human leukocyte antigen (HLA) match status, and donor-recipient cytomegalovirus (CMV) serostatus, were extracted from the Toronto Lung Transplant Database. As routine care, patients undergo routine surveillance bronchoscopy with transbronchial biopsies at 1.5, 3, 6, 9, and 12 months post-transplant. Daily home spirometry is also part of routine care and reviewed in clinic but not used in the adjudication of baseline values. Additional chart review was done for the _reversible drop_FEV_1_ and CLAD group. Of these 74 patients, 62 patients underwent bronchoscopy after FEV_1_ drop; 54 within 1 month, while 8 patients had bronchoscopies 1.5-2 months later. For the majority of patients, only abnormal secretions or mucosa were found. Bronchomalacia was observed in 2 patients in the _reversible drop_FEV_1_ group and 5 patients in the CLAD group. One CLAD patient had stenosis that had been noted prior to CLAD.

Baseline FEV_1_ was calculated first using the published definition of the average of the 2 best postoperative measurements taken at least 3 weeks apart (Supplemental Figure S1).^4^, ^12^ We then reviewed all available spirometry measurements after the baseline value was established to identify the patients who had a ≥20% drop in FEV_1_ from baseline. CLAD status was adjudicated according to the International Society for Heart and Lung Transplantation definition^3^ and after verification by chart review by a trained transplant pulmonologist.^13^ Patients, whose FEV_1_ recovered within 3 months after an initial a ≥20% drop, were categorized into the _reversible drop_FEV_1_ group. This group was identified by reviewing the spirometry data after the initial ≥20% drop; patients whose FEV_1_ improved to >80% of the baseline in the subsequent 3 months were considered to have reversible FEV_1_ drop. The earliest ≥20% drop was used for participants who experienced multiple drops (Supplemental Figure S1).

**Figure 1:**
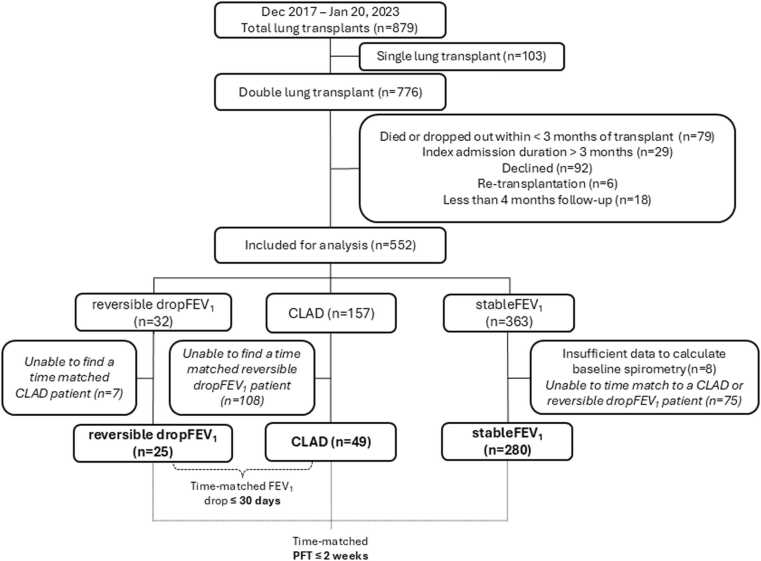
Patient recruitment, enrollment, and study cohort. Patient enrollment from December 2017 to January 2023 is shown. CLAD, chronic lung allograft dysfunction; FEV_1_, forced expiratory volume in 1 s; _reversible drop_FEV_1_, patients whose FEV_1_ recovered within 3 months after an initial a ≥20% drop; _stable_FEV_1_, patients whose FEV_1_ never dropped ≥20%.

Between December 2017 and January 2023, 776 of 879 LTx patients received double lung transplants (Figure 1). We excluded patients who died before 3 months post-transplant (n = 79), remained hospitalized at 3 months (n = 29), declined participation (n = 92), had retransplant (n = 6) or had <4 months follow-up (n = 18). Thus, 552 patients were included for analysis in this study. Patients who did not experience any drop in FEV_1_ during the study period were categorized into the _stable_FEV_1_ group (n = 363), 32 patients were identified as _reversible drop_FEV_1_ and 157 patients developed CLAD. Of the 32 _reversible drop_FEV_1_ patients, 25 were time-matched to 1-4 CLAD patient(s) whose CLAD onset date was within 30 days of the initial ≥20% FEV_1_ drop date of the _reversible drop_FEV_1_ patient (n = 49). We excluded 7 _reversible drop_FEV_1_ and 108 CLAD patients for whom we were unable to time-match to each other. The _reversible drop_FEV_1_ patients and their time-matched CLAD patient(s) were then time-matched to at least 2 _stable_FEV_1_ patients who had paired oscillometry-spirometry testing within 2 weeks of follow-up time from the date of transplant to create a _stable_FEV1 control group (n = 280) (Figure 1).

**Figure 2:**
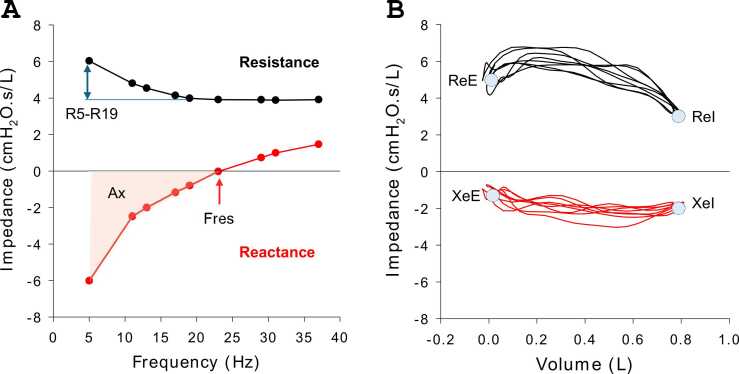
Key metrics of spectral oscillometry (A) and monofrequency (intrabreath) oscillometry (B). Resistance (R) and reactance (X) data are plotted in black and red lines, respectively. AX, reactance area between 5 Hz and Fres; Fres, resonance frequency; R5-R19, difference in resistance between 5 and 19 Hz; ReE, resistance at end expiration; ReI, resistance at end inspiration; XeE, reactance at end expiration; XeI, reactance at end inspiration.

The spectral oscillometry parameters of primary interest are^5^, ^10^: R5 (respiratory resistance at 5 Hz), which measures total lung resistance; R_5-19_ (difference in resistance from 5 to 19 Hz) a metric of small airway obstruction and ventilatory inhomogeneity; X5 (reactance of 5 Hz), a metric of the elastic properties of the lung; Fres (frequency of resonance), the frequency at which the elastic and inertial contributions are equal; and AX, the area of reactance between X5 and Fres (Figure 2A). X5 becomes more negative while AX and R5–19 increase with ventilatory inhomogeneity.^10^ Resistance (R) increases with progressive airway narrowing. Thus, the best respiratory mechanics are reflected by low R and high reactance (X) values. We used the same principle as spirometry for the calculation of the baseline (best) R and X values post-transplant. Intrabreath oscillometry parameters of interest include the R and X at end inspiration and end expiration (Figure 2B).^6^

We compared groups using one-way ANOVA for normally distributed variables, Kruskal-Wallis one-way analysis of variance for non-normal variables and Pearson’s chi-squared test for categorical variables. The data are shown as mean ± standard deviation (SD) or median and interquartile range (IQR) as appropriate. Pairwise comparisons between non-normally distributed variables were analyzed using Wilcoxon signed rank test with Bonferroni corrections. Conditional multivariable logistic regression analysis was used to assess the strength of associations between oscillometry parameters and CLAD. These were adjusted for biometric parameters of age at transplant, sex, and height, as they are associated with oscillometry measurements.^14^ Receiver operator characteristics (ROC) curves were used to determine the Youden index for oscillometry values to discriminate CLAD from _stable_FEV_1_ at FEV_1_ drop. The relative contributions of resistance and the reactance measurements were evaluated by dichotomizing each parameter as 1 if it fell into the CLAD-risk range or 0 if it fell with the FEV_1_ recovery range using the optimal cut-off values. Statistical analyses were performed using Prism 6.0 (GraphPad Software) and RStudio version 4.1.1 (The R Foundation).

## Results

### Demographics and baseline respiratory mechanics

Patient demographics and clinical characteristics, including donor-recipient CMV sero-status and donor-recipient HLA actual crossmatch status at time of transplant, GERD, and fundoplication were similar in CLAD, _reversible drop_FEV_1_, and _stable_FEV_1_ groups. The phenotypes of the CLAD patients were BOS (n = 26, 53.1%), RAS (n = 4, 8.2%), undefined (n = 2, 4.1%), and unclassified (n = 17, 34.6%). The most common indication for transplant was interstitial lung disease, followed by chronic obstructive pulmonary disease/emphysema, and cystic fibrosis/ bronchiectasis (Table 1). The duration of follow-up post-transplant, the best (i.e., baseline) FEV_1_ and baseline forced vital capacity (FVC) were similar among all 3 groups (Table 1). However, the _reversible drop_FEV_1_ and CLAD patients attained baseline spirometry earlier than the _stable_FEV_1_ patients. Baseline respiratory mechanics were different between the 3 groups. The highest X5 and lowest R5 values were found in the _stable_FEV_1_ groups. Lower X5 and higher R5 were seen in the _reversible drop_FEV_1_ and CLAD groups. In other words, the _stable_FEV_1_ groups had the best baseline respiratory mechanics. The time to achieve optimal respiratory mechanics occurred earlier than that for baseline spirometry for _stable_FEV_1_ (168.0 [81.0, 327.0] days) and CLAD (63.0 [38.0, 103.0] days) groups. Optimal respiratory mechanics were attained at 50-60 days (between 7 and 8 weeks) post-transplant, with no difference in the R5 among the 3 groups but worse (i.e., lower) X5 values in the CLAD group (Table 1, *p*<0.001).

**Table 1:** Patient Demographics at the Time of FEV_1_ Drop

	**CLAD**^**α**^	_**Reversible**__**drop**_**FEV**_**1**_	_**Stable**_**FEV**_**1**_	***p***
n	49	25	280	
Sex = M (*%*)	25 (51.0)	19 (76.0)	177 (63.2)	0.093
Age at Tx (*year*)	61.0 [47.0, 66.0]	65.00 [60.0, 69.0]	62.0 [55.8, 67.0]	0.111
Height at Tx (*cm*)	167.08 (8.48)	169.16 (10.40)	167.90 (9.34)	0.659
Weight at Tx (*kg*)	76.13 (14.85)	74.42 (14.30)	75.03 (16.53)	0.884
CMV D+/R-, n (*%*)	10 (20.4)	5 (20.0)	58 (20.7)	0.949
ACM +ve at Tx, n (*%*)	4 (8.2)	2 (8.0)	23 (8.2)	0.797
Primary Disease, n (*%*)				0.425
ILD	15 (30.6)	9 (36.0)	84 (30.0)	
COPD / Emphysema	11 (22.4)	7 (28.0)	105 (37.5)	
CF / Bronchiectasis	6 (12.2)	2 (8.0)	25 (8.9)	
Other	17 (34.7)	7 (28.0)	66 (23.6)	
GERD, n (%)	27 (55.1)	16 (64.0)	193 (68.9)	0.159
Fundoplication, n (%)	1 (2.0)	1 (4.0)	2 (0.7)	0.267
Baseline FEV_1_ (*L*)	2.56±0.80	2.31±0.57	2.53±0.79*	0.377
% FEV_1_	81.79±20.44	74.63±15.74	82.20±21.49**	0.227
Baseline FVC (*L*)	3.20±1.02	3.07±0.82	3.10±0.95*	0.763
% FVC	79.22±18.69	74.94±14.18	78.020±18.62**	0.636
Time to Baseline FEV_1_ (*days*)	63.0 [38.0, 103.0]	52.0 [36.0, 139.0]	168.0 [81.0, 327.0]**	**<0.001**
R5 baseline (cmH2O∙s/L)	3.03 [2.59, 3.46]	2.61 [2.11, 3.14]	2.75 [2.23, 3.45]	**0.029**
Time to R5 baseline (days)	51.96 [37.00, 77.04]	55.00 [34.00, 76.00]	50.00 [34.00, 79.25]	0.602
X5 baseline (cmH2O∙s/L)	−1.60 [-2.01, −1.29]	−1.28 [-1.61, −0.90]	−1.21 [-1.59, −0.93]	**<0.001**
Time to X5 baseline (days)	55.00 [37.96, 78.00]	61.00 [46.00, 138.00]	61.04 [42.00, 112.28]	0.167
Follow-up (*days*)	230.0 [165.0, 325.0]	200.0 [108.0, 299.0]	191.0 [100.0, 313.0]	0.448

Continuous normal variables are reported as mean (SD); non-normal variables as median [IQR]

^α^The CLAD phenotypes are BOS (n = 26, 53.1%), RAS (n = 4, 8.2%), undefined (n = 2, 4.1%), and unclassified (n = 17, 34.6%). Follow-up time is calculated to FEV_1_ drop; or time matched to FEV_1_ drop for the _stable_FEV_1_ patients

One-way ANOVA was used to compare parametric continuous parameters. Kruskal-Wallis test was used to compare non-parametric continuous parameters. Chi-squared test or Fisher’s exact test was used to compare categorical variables

ACM, actual cross match; CF, cystic fibrosis; CMV, cytomegalovirus; COPD, chronic obstructive lung disease; D, donor; FEV_1_, forced expiratory volume in 1 s; FVC, forced vital capacity; GERD, gastroesophageal reflux disease; ILD, interstitial lung disease; R, recipient; Tx, transplant

* Missing n = 5

** Missing n = 20

### Spirometry, plethysmography, and oscillometry at initial >20% FEV_1_ drop and in _stable_FEV_1_ patients at the time-matched point after transplant

Significant differences in spirometry and plethysmography parameters were observed between the 3 groups at time of the initial FEV_1_ drop for the CLAD and _reversible drop_FEV_1_ groups, and at the time-matched point in the _stable_FEV_1_ patients, (Table 2). CLAD patients had the lowest percent predicted (%) values of FEV_1_, FVC, and TLC values among the 3 groups, as well as the highest RV (residual volume)/TLC (total lung capacity) ratio, suggesting gas-trapping (Table 2). Post hoc analysis revealed that the significant differences were primarily due to differences between the _stable_FEV_1_ vs _reversible drop_FEV_1_ groups, and the _stable_FEV_1_ vs CLAD groups (Table 2). No spirometry nor plethysmography parameters were significantly different between CLAD vs _reversible drop_FEV_1_ groups.

**Table 2:** Standard Pulmonary Function Metrics at the Time of ≥ 20% FEV_1_ Drop

	**CLAD**	_**Reversible**__**drop**_**FEV**_**1**_	_**Stable**_**FEV**_**1**_	***p***
**FEV**_**1**_**(***L***)**	1.49±0.54ⱡ	1.70±0.41δ	2.41±0.79ⱡδ	**<0.001**
% FEV_1_	48.03±15.38ⱡ*	54.96±12.10δ*	77.67±21.63ⱡδ	**<0.001**
**FVC (***L***)**	2.36±0.83ⱡ	2.64±0.75δ	3.01±0.92ⱡδ	**<0.001**
% FVC	60.08±17.69ⱡ	64.23±11.99δ	75.42±18.16ⱡδ	**<0.001**
**FEV**_**1**_**/FVC (***%***)**	65.20 [54.30, 69.50]ⱡ	63.80 [60.00, 70.20]δ	80.45 [73.68, 89.10]ⱡδ	**<0.001**
% FEV_1_/FVC	82.90 [69.40, 90.60]ⱡ	81.20 [75.10, 92.30]δ	104.15 [95.27, 112.82]ⱡδ	**<0.001**
**TLC (***L***)**	4.31±1.15ⱡ	4.59±1.10	4.87±1.13ⱡ	**0.009**
% TLC	70.32±14.01ⱡ	72.22±15.50^δ^	78.96±15.27ⱡδ	**0.001**
**RV (***L***)**	1.79±0.51	1.86±0.58	1.80±0.55	0.859
% RV	90.96±26.69	86.24±29.78	87.69±29.84	0.763
**RV/TLC (***%***)**	42.31±8.75ⱡ	40.58±8.09	37.48±9.31ⱡ	**0.003**
% RV/TLC	115.86±28.98ⱡ	104.46±20.46	98.98±26.94ⱡ	**0.001**
**DLCO (***mL/min/mmHg***)**	12.82±4.07ⱡ	12.90±4.79	16.00±4.84ⱡ	**0.019**
% DLCO	68.87±15.77	68.66±12.70	77.80±15.96	0.052

*p*<0.05 for pairwise comparisons as indicated

Continuous normal variables are reported as mean (SD); non-normal variables as median [IQR]. Statistics were performed using one-way ANOVA or Kruskal-Wallis test for parametric or non-parametric parameters, respectively. For pairwise comparisons, Student’s t-test or Wilcoxon rank-sum test with Bonferroni correction for multiple comparisons was conducted for parametric or non-parametric parameters, respectively

DLCO, diffusing capacity for carbon monoxide; FEV_1_, forced expiratory volume in 1 s; FVC, forced vital capacity; RV, residual volume; TLC, total lung capacity

ⱡ CLAD vs _stable_FEV_1_

* CLAD vs _reversible drop_FEV_1_ indicate

δ _reversible drop_FEV_1_ vs _stable_FEV_1_

**Figure 3:**
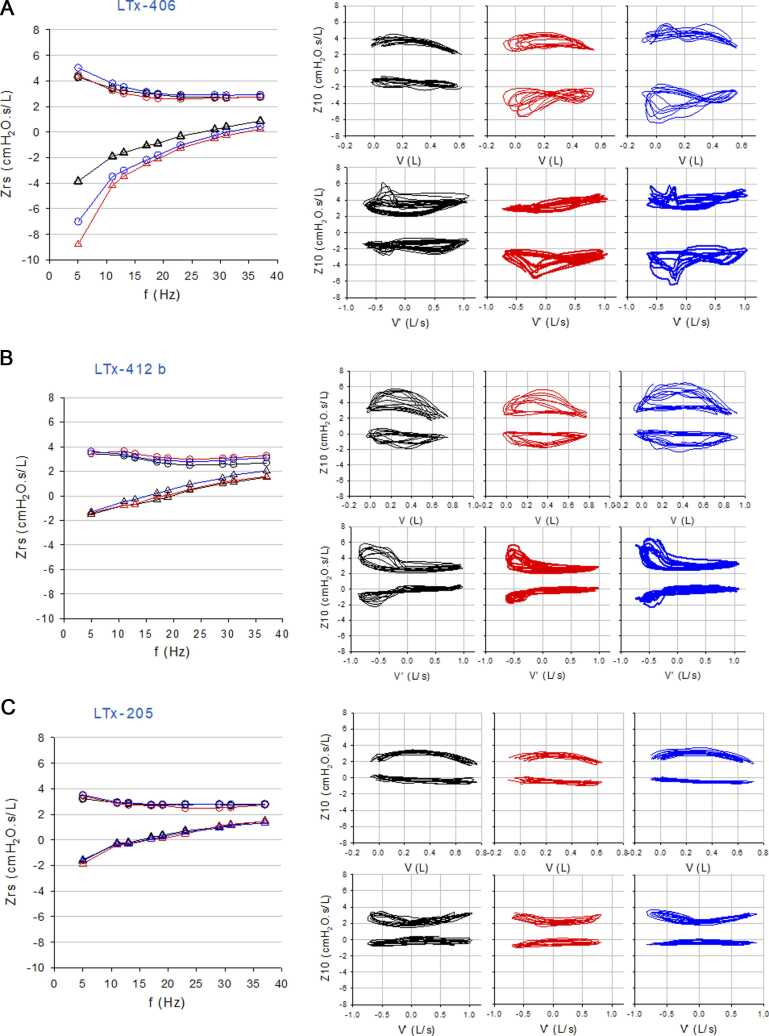
Representative spectral (left) and intrabreath (right) oscillograms of a patient (A) who developed CLAD, (B) whose FEV_1_ recovered (_reversible drop_FEV_1_), and (C) who did not experience any ≥20% drops in FEV_1_ (_stable_FEV_1_). Resistance (circles) and reactance (triangles) were plotted at baseline (black), FEV_1_ drop (red), and the first visit 3 months or more after the FEV_1_ drop (blue). The oscillograms in the _reversible drop_FEV_1_ patient (B) were similar at all 3 time points, in contrast to the CLAD patient (A) where reactance curve at initial FEV_1_ drop was downward and rightward shifted compared to baseline.

All spectral oscillometry and most intrabreath oscillometry parameters were significantly different among the 3 groups (Table 2). Oscillometry at time of the initial FEV_1_ drop revealed differences in lung mechanics between _reversible drop_FEV_1_ and CLAD patients. The CLAD group had worse lung mechanics with higher R5, AX, Fres, and more negative X5. Representative oscillograms at baseline spirometry, initial FEV_1_ drop and 12 weeks later are shown in Figure 3A-C. Notably, the oscillograms in the _reversible drop_FEV_1_ patient (panel B) were similar at all 3 time points compared to the CLAD patient (Figure 3A), who showed a drop in reactance compared to baseline. Intrabreath oscillometry also revealed progressively worsening mechanics from the _stable_FEV_1_, _reversible drop_FEV_1_ to the CLAD groups with the CLAD patients having the highest ReE and ReI, and most negative XeE and XeI values. The _stable_FEV_1_ patients who did not experience any FEV_1_ drops had the best spectral and intrabreath oscillometry measurements. Post hoc paired comparisons showed that most of the observed differences were between CLAD vs _stable_FEV_1_ groups, and the _reversible drop_FEV_1_ drop vs _stable_FEV_1_ groups. Among the oscillometry parameters, X5 (Bonferroni adjusted *p* = 0.0287) was significantly different between CLAD and _reversible drop_FEV_1_ patients (Table 2).

### Oscillometry at FEV_1_ drop is associated with CLAD

We investigated the relationship between the oscillometry parameters at time of initial FEV_1_ drop and subsequent development of CLAD (Table 3). These analyses focused on the 74 time-matched patients in the _reversible drop_FEV_1_ and CLAD groups. X5 and X5 z-score were associated with CLAD. For each 1 unit drop in X5, the odds of developing CLAD within 3 months of the initial FEV_1_ drop was 90% higher (95% CI: 1.01-3.57). While R5-19 was not significantly associated with CLAD, every unit increase in R5-19 was associated with 117% higher (95% CI: 0.91-5.16) odds of developing CLAD within 3 months of the FEV_1_ drop. We assessed for potential confounding effects of clinical factors and found age at transplant to be the primary confounder of the associations between spectral oscillometry parameters (X5, X5 z-score, and R5-19) and CLAD (Supplementary Table 1).

**Table 3:** Spectral and Oscillometry Metrics at the Time of ≥ 20% FEV_1_ Drop

**Spectral oscillometry**	**CLAD**	_**Reversible**__**drop**_**FEV**_**1**_	_**Stable**_**FEV**_**1**_	***p***
**R5**^*a*^	4.31 [3.62, 4.93]ⱡ	3.53 [3.06, 5.17]	3.45 [2.72, 4.42]ⱡ	**<0.001**
**R5 Z-score**	1.07 (1.07)ⱡ	1.27 (1.09)δ	0.5 (1.20)ⱡδ	**0.001**
**R**_**5–19**_^*a*^	0.96 [0.70, 1.61]ⱡ	0.96 [0.57, 1.30]δ	0.55 [0.24, 1.01]ⱡδ	**<0.001**
**X5**^*a*^	−2.77 [-3.89, −2.16]ⱡ*	−1.98 [−2.80, −1.56]δ*	−1.51 [−2.12, −1.15]ⱡδ	**<0.001**
**X5 Z-score**	−3.95(2.77)ⱡ*	−2.62 (1.78)δ*	−1.29 (2.10)ⱡδ	**<0.001**
**AX**^*b*^	20.59 [13.77, 33.76]ⱡ	14.65 [9.92, 22.81]δ	8.36 [4.96, 13.12]ⱡδ	**<0.001**
**Fres (***Hz***)**	23.20 [21.17, 26.90]ⱡ	20.53 [19.45, 24.96]δ	17.75 [14.26, 20.72]ⱡδ	**<0.001**
**Intrabreath Oscillometry**	**CLAD**	_**reversible drop**_**FEV**_**1**_	_**stable**_**FEV**_**1**_	**p**
**ReE**^*a*^	3.86 [3.08, 4.15]ⱡ	3.08 [2.62, 4.46]δ	2.84 [2.26, 3.66]ⱡδ	**<0.001**
**ReI**^*a*^	2.64 [2.17, 3.03]ⱡ	2.35 [2.00, 2.98]	2.29 [1.92, 2.77]ⱡ	**0.009**
**XeE**^*a*^	−1.12 [−2.21, −0.47]ⱡ	−0.67 [−1.60, −0.40]δ	−0.17 [−0.60, 0.08]ⱡδ	**<0.001**
**XeI**^*a*^	−0.84 [−1.14, −0.49]ⱡ*	−0.44 [−0.85, −0.29]δ*	−0.27 [−0.58, −0.07]ⱡδ	**<0.001**

Units of measure: ^*a*^ cmH_2_O∙s/L; ^*b*^ cmH_2_O/L

*p*<0.05 for pairwise comparisons as indicated

Statistics were performed with Kruskal-Wallis one-way analysis of variance. Pairwise comparisons were conducted using Wilcoxon signed rank test with Bonferroni correction for multiple comparisons

Ax, reactance area between 5 Hz and Fres; Fres, resonance frequency; R5, resistance at 5 Hz; R19: resistance at 19 Hz; R_5-19_, difference in resistance between 5 and 19 Hz; ReE, resistance at end-expiration; ReI, resistance at end-inspiration; X5, reactance at 5 Hz; XeE, reactance at end-expiration; XeI, reactance at end-inspiration

ⱡ CLAD vs _stable_FEV_1_

δ _reversible drop_FEV_1_ vs _stable_FEV_1_

* CLAD vs _reversible drop_FEV_1_

The optimal threshold values for the spectral and intrabreath oscillometry that would discriminate against future CLAD from _reversible drop_FEV_1_ were determined using ROC curves (Supplementary Figure 3). X5 had the highest area under the curve (AUC: 0.703), followed by XeI (0.664) and Fres (0.576) (Table 4). X5 and ReE exhibited the highest sensitivity (0.82). XeE has the highest specificity (0.88) followed by R5–19 (0.84) (Table 4).

**Table 4:** Associations of Oscillometry Metrics at FEV_1_ Drop With CLAD

	**Unadjusted Models**		**Adjusted Models**	
	**OR (95% CI)**	***p*****value**	**OR (95% CI)**	***p*****value**
**Spirometry**				
**% FEV**_**1**_	0.96 (0.93-1.00)	0.065	NA	NA
**Spectral Oscillometry**				
**R5**^*a*^	1.22 (0.81-1.85)	0.347	1.32 (0.82-2.12)	0.248
**R5 z-score**	0.97 (0.61-1.52)	0.879	1.02 (0.59-1.76)	0.933
**R5–19**^*a*^	1.40 (0.74-2.67)	0.304	2.17 (0.91-5.16)	0.081
X5*^a^	1.65 (1.04-2.62)	**0.033**	1.90 (1.01-3.57)	**0.046**
**X5 z-score**	0.78 (0.61-0.99)	**0.044**	0.72 (0.53-0.98)	**0.039**
**AX**^*b*^	1.03 (0.99-1.07)	0.134	1.03 (0.99-1.08)	0.147
**Fres (***Hz***)**	1.03 (0.93-1.13)	0.594	1.07 (0.94-1.20)	0.312
**Intrabreath Oscillometry**				
**ReE**^*a*^	1.16 (0.67-2.01)	0.588	1.16 (0.60-2.25)	0.670
**ReI**^*a*^	1.56 (0.68-3.59)	0.292	1.83 (0.65-5.20)	0.256
XeE*^a^	1.36 (0.87-2.12)	0.174	1.47 (0.86-2.53)	0.159
XeI*^a^	2.66 (0.96-7.42)	0.061	2.99 (0.82-10.95)	0.097

Conditional univariable and multivariable logistic regression analysis were used. Multivariable model consisted of adjustments for age, sex, and height

Units of measure: ^*a*^ cmH_2_O∙s/L; ^*b*^ cmH_2_O/L

* Reactance metrics were multiplied with ‘-1’ before conditional logistic regression analysis

Lastly, we evaluated the contribution of the resistance and reactance to the risk assessment of progression to CLAD or FEV_1_ recovery at time of initial FEV_1_ drop. R5 and X5 were included in these models as ROC analysis revealed the highest AUCs and sensitivities amongst the different oscillometry parameters (Table 4). Of the 74 patients with FEV_1_ drop to ≤80% of baseline, oscillometry revealed that 16 patients had both R5 and X5 in the FEV_1_ recovery range, 41 patients had both R5 and X5 in the CLAD range, and 17 patients had either R5 or X5 in the CLAD range. When both R5 and X5 fell in the CLAD-risk range at the time of FEV_1_ drop, there were 1163% (95% CI: 2.93-69.26) greater odds for CLAD than those who did not, with a large CI most likely due to small sample size (Table 5).

**Table 5:** Cut Off Values for Spectral and Intrabreath Oscillometry Parameters

**Spectral Oscillometry**	**AUC**	**Optimal Cutoff**	**Sensitivity**	**Specificity**
**R5**^*a*^	0.612	3.618	0.76	0.56
**R**_**5-19**_^*a*^	0.551	1.368	0.39	0.84
**X5**^*a*^	0.703	-2.025	0.82	0.56
**AX**^*b*^	0.620	14.920	0.65	0.60
**Fres (***Hz***)**	0.576	20.854	0.76	0.52
**Intrabreath Oscillometry**	**AUC**	**Optimal Cutoff**	**Sensitivity**	**Specificity**
**ReE**^*a*^	0.574	2.935	0.82	0.48
**ReI**^*a*^	0.558	2.453	0.61	0.60
**XeE**^*a*^	0.589	-1.800	0.39	0.88
**XeI**^*a*^	0.664	-0.489	0.76	0.60

ROC were used to determine the optimal cut-off values to discriminate CLAD from _stable_FEV_1_ at FEV_1_ drop

## Discussion

There is no effective medical treatment for CLAD, making prevention and early diagnosis essential to its management. There is broad consensus that pulmonary function tests and imaging modalities are re--ed for diagnosing, phenotyping, and monitoring CLAD.^15^ Invasive procedures such as transbronchial biopsy are also used to rule out potentially reversible processes such as acute rejection and infection. While spirometry is widely regarded as essential for the assessment and follow-up of CLAD, body plethysmography is less accessible, and results were unavailable in 16% of cases in a European survey.^15^ This highlights the advantages of oscillometry, which is non-invasive, easier to perform, portable, and offers additional diagnostic information. This is the first study to assess LTx patients with oscillometry at time of the initial ≥20% drop in FEV_1_ to determine whether oscillometry could provide additional information that can help distinguish between patients who will subsequently develop CLAD from patients whose FEV_1_ will recover. We found that reactance measurement, X5, was the most strongly associated with development of CLAD.

The demographic and peri-operative characteristics, and duration of follow-up were similar for all 3 groups. The CLAD and _reversible drop_FEV_1_ groups attained baseline FEV_1_ earlier than the _stable_FEV_1_ patients although there were no significant differences in the baseline values between the 3 groups. In contrast, the baseline (or best) oscillometry values were achieved earlier than spirometry, at 7-8 weeks post-transplant, and similar for all 3 groups. Moreover, the CLAD group exhibited the worst baseline respiratory mechanics, as shown by lower X5 and higher R5 values (Table 1). These observations suggest that optimal lung mechanics are already established by 2 months post-transplant and improvements in spirometry beyond this time point likely relate to extra-pulmonary factors such as chest wall remodeling, improved diaphragm and respiratory muscle strength and overall physical conditioning.

At time of FEV_1_ drop, both spirometry and oscillometry showed obstructive patterns in the CLAD and _reversible drop_FEV_1_ groups, i.e., decreased FEV_1_/FVC, increased R5-19, increased AX and low X5.^16^, ^17^ No differences in FEV_1_/FVC were found between the CLAD and _reversible drop_FEV_1_ groups. In contrast, oscillometry revealed significantly worse respiratory mechanics in the CLAD group with lower X5, X5 z-score and XeI (Table 2). Patients with lower X5 at time of the FEV_1_ drop had a higher risk for progression to CLAD rather than recovery (Table 3). Resistance and reactance reflect different aspects of respiratory mechanics. Thus, we evaluated the contributions R5 and X5 with respect to their effect on association with CLAD. When both R5 and X5 fell in the CLAD-risk range, the OR of progression to CLAD within 12 weeks was greater than when either R5 or X5 fell in the CLAD-risk range or when neither R5 nor X5 fell in the CLAD-risk range (Table 5). In other words, patients with the worse respiratory mechanics (higher R5 and lower X5 values) at time of FEV_1_ drop were at highest risk of CLAD. These findings highlight the sensitivity of oscillometry for detecting graft dysfunction that are indiscernible by spirometry and that it can do so significantly earlier than standard pulmonary function test such as spirometry and plethysmography.

Our sample size was limited by the fact that most lung transplant patients undergo pulmonary function testing infrequently at our center beyond 3 months post-transplant, when the majority experience a ≥20% drop in their FEV_1_. Furthermore, we used time-matching of the _stable_FEV_1_ patients to the date of the initial FEV_1_ drop of the CLAD and _reversible drop_FEV_1_ patients to account for the time-varying changes in lung function and respiratory mechanics amongst the patients. However, this strategy reduced the overall sample size as 108 CLAD patients and 7 _reversible drop_FEV_1_ patients were excluded from analysis because corresponding time-matched _stable_FEV_1_ patients could not be found in the study cohort. Oscillometry is also highly dependent on proper technique to ensure accurate, high-quality measurements.^11^To minimize lapses in quality control amongst the 20 operators responsible for oscillometry testing during the study period, all staff were trained and followed the same standard operating protocol with biweekly quality control audits of all oscillometry tests. An important aspect of the study is that patients were transplanted at one center and were evaluated using the same oscillometry device. Systemic differences in the measurements, particularly of reactance, exist amongst the different commercially available oscillometers.^4^, ^5^, ^10^, ^18^ While a single centre, single device allows for consistent measurements, it may not be applicable to other centers. As such, it will be important to validate for our findings in a larger patient population, in another center and with different commercial devices.

## Conclusion

Our findings add to the growing evidence of the high sensitivity of oscillometry when compared to spirometry with respect to relevant clinical outcomes in different lung diseases.^7^, ^8^, ^9^, ^18^^19^ This study in lung transplant recipients revealed significant differences in oscillometry at time of the initial 20% drop in FEV_1_ between patients who subsequently developed CLAD from those whose FEV_1_ recovered within 3 months. The addition of oscillometry to spirometry as part of routine monitoring after LTx could facilitate earlier identification of CLAD, i.e., at time of the initial drop of FEV_1_ to <80% of baseline. This would prompt earlier investigations and interventions. As new therapies become available for the management of CLAD, oscillometry at time of FEV_1_ drop could also allow for earlier enrollment of patients into clinical trials before irreversible damage occurs by waiting 12 weeks after the initial FEV_1_ drop.

## CRediT authorship contribution statement

A.F: conducted the data organization, helped develop the statistical analysis plan, conducted the statistical analysis and drafted the manuscript. A.V: cleaned the clinical database, verified clinical data, and edited the manuscript. N.H: developed the statistical plan and verified the initial analysis. N.B: verified clinical data and edited the manuscript. J.K.Y.W: maintained the research ethics protocol, developed the standard operating procedures and quality control of the oscillometry tests, and quality assurance of the data. E.H. oversaw all aspects of the statistical analysis. Z.H. developed the intrabreath technique and oversaw the quality control and assurance of all intrabreath analysis and data. C-W.C: developed the concept, study protocol and oversaw all aspects of the project.

## Disclosure statement

The authors declare no conflict of interest related to this study. CWC received investigator-initiated research funding and speaking fees from Thorasys Thoracic Medical Device Corp and speaking fees from AstraZeneca Incorporated and Sanofi Ltd. J.K.Y.W has received speaking fees from Thorasys Thoracic Medical Device Corp and Sanofi.

## Declaration of competing interest

The authors declare that they have no known competing financial interests or personal relationships that could have appeared to influence the work reported in this paper.

## Footnotes

Appendix A Supplementary data associated with this article can be found in the online version at doi:10.1016/j.jhlto.2026.100575.
