# Supplementary material for: Early detection of chronic lung allograft dysfunction with spectral and intrabreath oscillometry
Source: JHLT Open. 2026 May 4;13:100575. doi: 10.1016/j.jhlto.2026.100575 (PMC13235334; doi:10.1016/j.jhlto.2026.100575)
Supplement: Supplementary file 1 — Supplementary material [file mmc1.docx]

**Spectral and Intrabreath Oscillometry for Early Diagnosis of Chronic Lung Allograft**

**Dysfunction**

**SUPPLEMENTAL MATERIAL**

Anne Fu^1^ <https://orcid.org/0000-0003-3886-1853>

Anastasiia Vasileva^1^ <https://orcid.org/0000-0002-5915-9265>

Nour Hanafi^1^ <https://orcid.org/0000-0003-2922-705X>

Natalia Belousova^4^ <https://orcid.org/0000-0002-2237-4172>

Joyce K. Y. Wu^1,2^ [https://orcid.org/0000-0002-4113-2531](https://urldefense.com/v3/__https:/orcid.org/0000-0002-4113-2531__%3B!!CjcC7IQ!M9O4eWzb4w0zMbP6pi_SpJWxc2cURSH9Jm_cjrTiCsApMwICmzseubTenGhnlDfbRpbjGTOScQbJAeN5Sf6AkYPzcU--YNPd$)

Ella Huszti^5^ <https://orcid.org/0000-0003-0169-4895>

Zoltán Hantos^6^ <https://orcid.org/0000-0002-5696-7750>

Chung-Wai Chow^1,2,3^ <https://orcid.org/0000-0001-9344-8522>

1. Department of Medicine, Temerty Faculty of Medicine, University of Toronto, Toronto, ON, Canada.

2. Pulmonary Function Laboratory, University Health Network, Toronto, ON, Canada.

3. Toronto Lung Transplant Program, Ajmera Multi-Organ Transplant Unit, University Health Network, Toronto, ON, Canada.

4. Thoracic Medicine and Lung Transplant Unit, St Vincent’s Hospital, Sydney, Australia

5. Biostatistics Research Unit, University Health Network, Toronto, ON, Canada.

6. Department of Anesthesiology and Intensive Therapy, Semmelweis University, Budapest, Hungary.

**
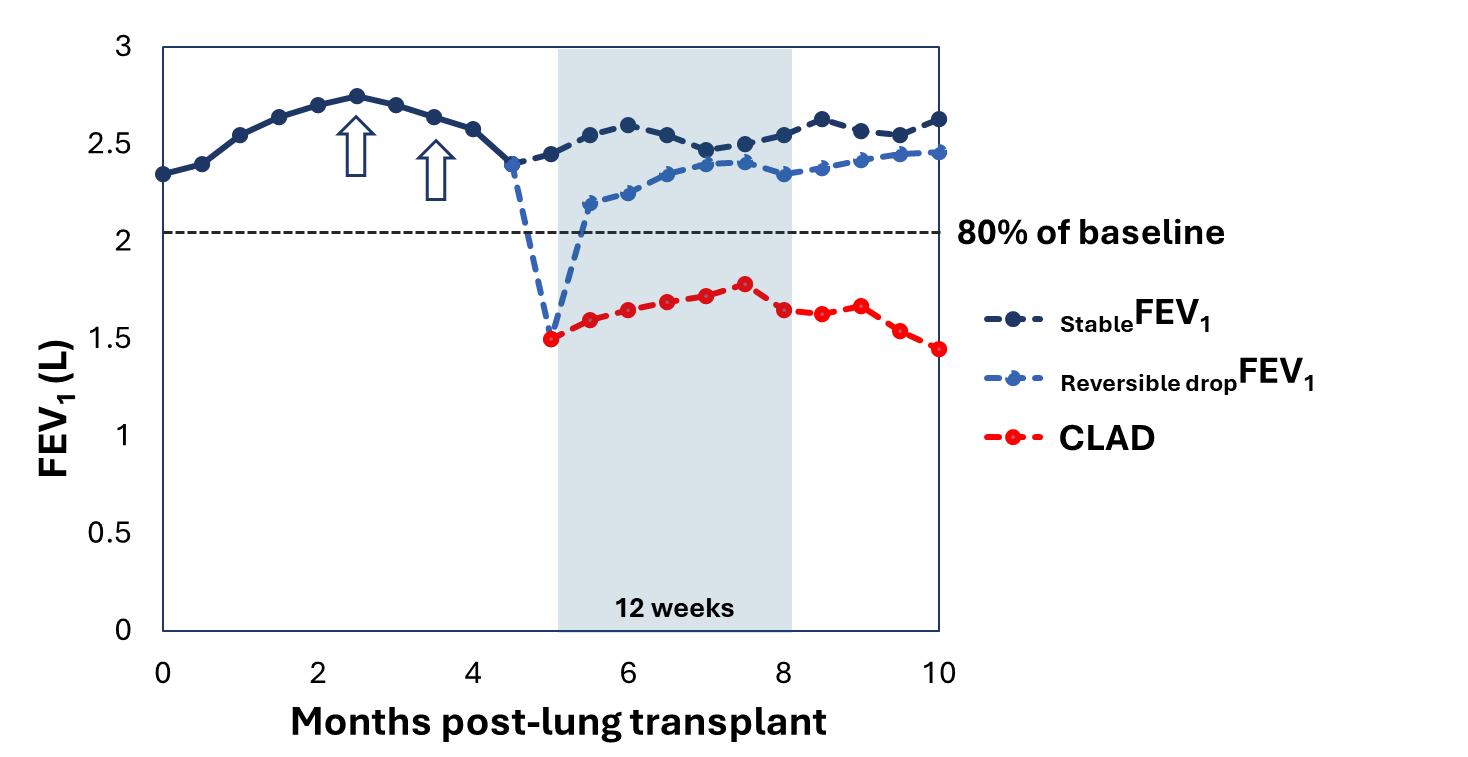
**

**Figure S1. Graph depicting the changes in FEV_1_ over time post-lung transplant.** Baseline FEV_1_ was calculated from the average of the two best postoperative measurements taken at least 3 weeks apart (indicated by the arrows). Patients who had a ≥20% drop in FEV_1_ below the 80% threshold and did not recover within 3 months had CLAD. Patients whose FEV_1_ recovered within 3 months after an initial a ≥20% drop were categorized into the _Reversible drop_FEV_1_ group. Lastly, patients whose FEV_1_ never dropped were in the _Stable_FEV_1_ group.

**
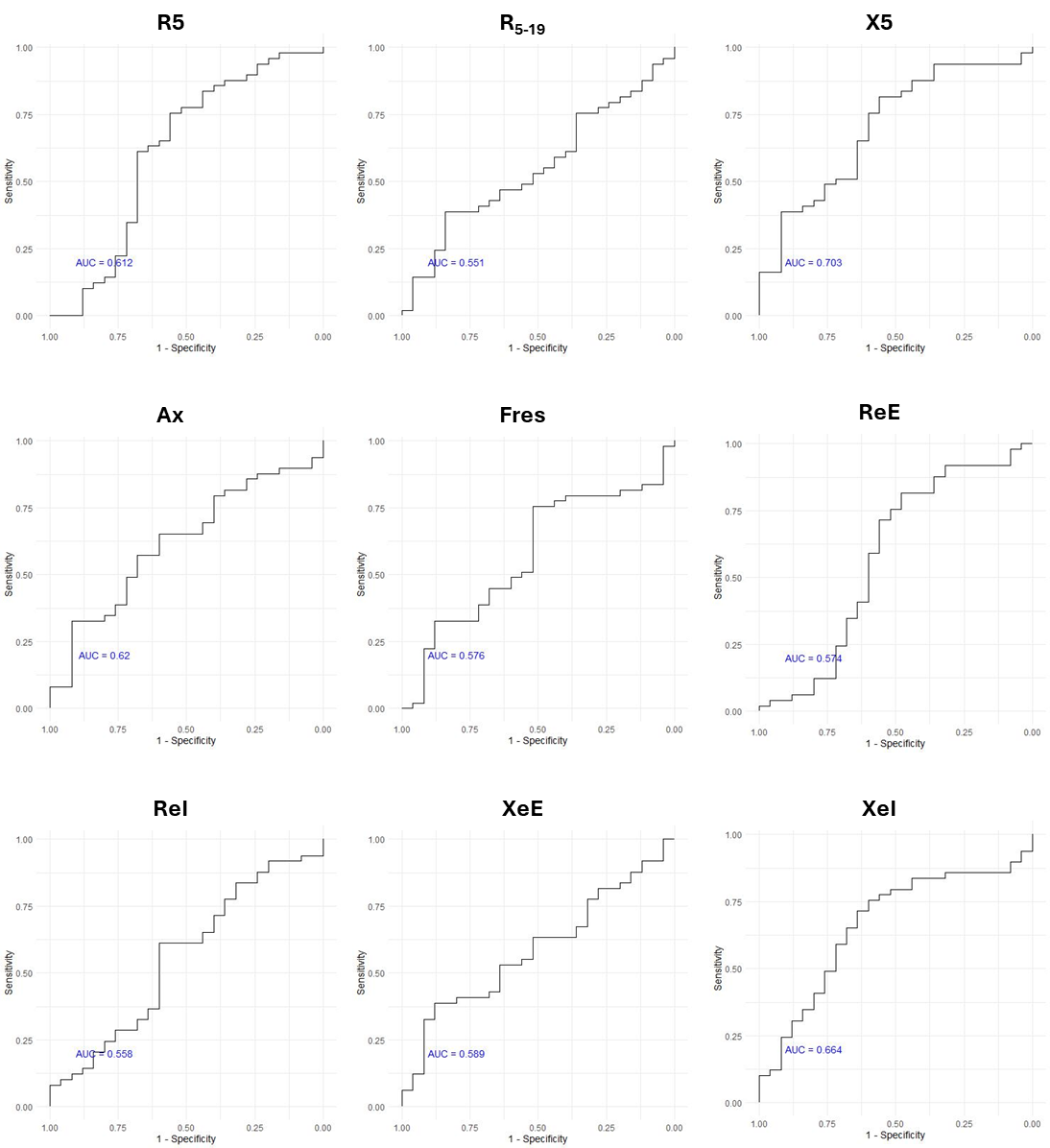
**

**Figure S2.** **Receiver operator curves and area under the curves for the spectral and intrabreath oscillometry parameters for differentiating CLAD from _Reversible drop_FEV_1_ at FEV_1_ drop.**

**Table S1.** Bivariable models of confounding variables sex, height, or age at transplant with oscillometry parameters X5, X5 z-score, and R5-19.

| **Confounding Variable** | **Parameters** | **OR (95% CI)** | ***P* value** |
| --- | --- | --- | --- |
| **Age** | **X5 (incr.)** | 2.01 (1.10 – 3.66) | **0.022** |
|  | **Age** | 0.94 (0.89 – 0.99) | **0.026** |
|  | **X5 z-score (decr.)** | 0.72 (0.54 – 0.95) | **0.021** |
|  | **Age** | 0.94 (0.89 – 0.99) | **0.029** |
|  | **R5-19 (incr.)** | 2.13 (0.95 – 4.78) | 0.066 |
|  | **Age** | 0.93 (0.88 – 0.99) | **0.023** |
| **Sex (Male vs. Female)** | **X5 (decr.)** | 1.51 (0.94 – 2.42) | 0.086 |
|  | **Sex** | 0.47 (0.14 – 1.60) | 0.227 |
|  | **X5 z-score (incr.)** | 0.79 (0.62 – 1.01) | 0.062 |
|  | **Sex** | 0.35 (0.11 – 1.15) | 0.083 |
|  | **R5-19** | 1.40 (0.70 – 2.78) | 0.339 |
|  | **Sex** | 0.32 (0.10 – 1.05) | 0.060 |
| **Height** | **X5 (decr.)** | 1.63 (1.02 – 2.60) | **0.040** |
|  | **Height** | 0.99 (0.93 – 1.05) | 0.684 |
|  | **X5 z-score (decr.)** | 0.76 (0.59 – 0.98) | **0.035** |
|  | **Height** | 0.97 (0.91 – 1.02) | 0.246 |
|  | **R5-19 (incr.)** | 1.43 (0.74 – 2.76) | 0.292 |
|  | **Height** | 0.98 (0.92 – 1.03) | 0.375 |
